# Immunoinformatic-Based Prediction of Candidate Epitopes for the Diagnosis and Control of Paratuberculosis (Johne’s Disease)

**DOI:** 10.3390/pathogens9090705

**Published:** 2020-08-27

**Authors:** Bruno Tilocca, Alessio Soggiu, Viviana Greco, Cristian Piras, Norma Arrigoni, Matteo Ricchi, Domenico Britti, Andrea Urbani, Paola Roncada

**Affiliations:** 1 Department of Health Science, University “Magna Græcia” of Catanzaro, Viale Europa, 88100 Catanzaro, Italy; tilocca@unicz.it (B.T.); c.piras@unicz.it (C.P.); britti@unicz.it (D.B.); 2 Department of Biomedical, Surgical and Dental Sciences—One Health Unit, University of Milano, Via Celoria10, 20133 Milano, Italy; alessio.soggiu@unimi.it; 3 Department of Veterinary Medicine, University of Milano, Via dell’Università 6, 26900 Lodi, Italy; 4 Department of Basic Biotechnological Sciences, Intensivological and Perioperative Clinics, Università Cattolica del Sacro Cuore, Largo Francesco Vito 1, 00168 Roma, Italy; viviana.greco@unicatt.it (V.G.); andrea.urbani@unicatt.it (A.U.); 5 Dipartimento di Scienze di laboratorio e infettivologiche, Fondazione Policlinico Universitario Agostino Gemelli IRCCS, Largo A. Gemelli 8, 00168 Roma, Italy; 6 National Reference Center for Paratuberculosis, Istituto Zooprofilattico Sperimentale della Lombardia e dell’Emilia Romagna, Strada Faggiola 1, 29027 Gariga di Podenzano, Italy; norma.arrigoni@izsler.it (N.A.); matteo.ricchi@izsler.it (M.R.)

**Keywords:** paratuberculosis, proteomics, *Mycobacterium avium* subsp. *paratuberculosis* (MAP), immunoinformatics, early diagnosis, vaccines, epitopes

## Abstract

Paratuberculosis is an infectious disease of ruminants caused by *Mycobacterium avium* subsp. *paratuberculosis* (MAP). MAP is an intracellular pathogen with a possible zoonotic potential since it has been successfully isolated from the intestine and blood of Crohn’s disease patients.Since no cure is available, after the detection of the disease, animal culling is the sole applicable containment strategy. However, the difficult detection of the disease in its subclinical form, facilitates its spread raising the need for the development of effective diagnosis and vaccination strategies. The prompt identification and isolation of the infected animals in the subclinical stage would prevent the spread of the infection.In the present study, an immunoinformatic approach has been used to investigate the immunogenic properties of 10 MAP proteins. These proteins were chosen according to a previously published immunoproteomics approach. For each previously-described immunoreactive protein, we predicted the epitopes capable of eliciting an immune response by binding both B-cells and/or class I MHC antigens. The retrieved peptide sequences were analyzed for their specificity and cross-reactivity. The final aim is to employ the discovered peptides sequences as a filtered library useful for early-stage diagnosis and/or to be used in novel multi-subunit or recombinant vaccine formulations.

## 1. Introduction

Bovine paratuberculosis, also known as Johne’s disease (JD) is an infectious disease of ruminants caused by *Mycobacterium avium* subsp. *paratuberculosis* (MAP). It is characterized by chronic and progressive granulomatous enteritis. The infected animals initially show normal appetite and food consumption, but the intestinal wall thickening and the impaired nutrient absorption cause a reduced feed-conversion rate and a progressive weight loss. Milk yield is also negatively affected by the progression of the infection. Nevertheless, clinical manifestations do not involve all infected animals [1,2,3]; the subclinical stageof infection can last from 2 to 15 years [4] and, despite the absence of visible symptoms, animals in this stage can shed MAP and spread the disease [3,5,6,7]. For these aforementioned reasons, this pathology leads to significantly increased veterinary costs worldwide [3,8,9].

The causal agent of JD is MAP. It is considered a zoonotic pathogen [10] because of its possible link with Crohn’s disease. MAP infection affects animals and there is considerable evidence that might be a co-cause of human Crohn’s disease [11]. MAP isolation from the intestine and blood of Crohn’s disease patients has extensively documented. More precisely, MAP presence was found to be seven times higher in Crohn’s disease patients than what has been found in patients with any other bowel inflammation [12,13]. MAP also infected animals and Crohn’s disease patients show similar alterations of the immune system response reinforcing the hypothesis about the analogy between the two [14,15,16,17].

MAP is a slow-growing bacterium, commonly acquired via the fecal-oral route by both animals and humans [18]. Despite the pathogenetic mechanism of MAP, infection has not been fully understood, it has been demonstrated that its acid resistance enables it to survive in the gastric environment, allowing its entrance in the intestinal tract. At the ileal level, MAP invades the lymphatic system overlying Peyer’s patches. This stimulates the host’s immune response that, besides activating the humoral response, promptly phagocytizes MAP into macrophages [8]. As an intracellular pathogen, MAP can either survive into macrophagic cells or being killed and disassembled to present its antigens to T-lymphocytes [3].

**Figure 1:**
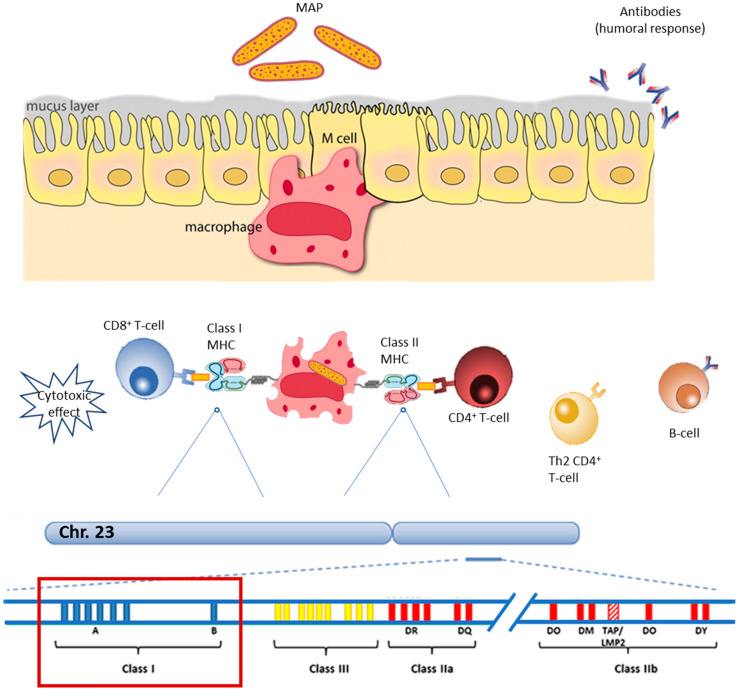
Schematic overview of the *Mycobacterium avium* subsp. *paratuberculosis* (MAP) immunity.

Evidence from multibacillary JD revealed a massive humoral antibody response along with a tendency to suppress the cell-mediated immune response [3,19,20]. Whereas, a recent comparative study between two groups of cows, one in the sub-clinical and the other in the clinical stage, highlighted an increased T-cell activity in the first group of animals compared to the second one [21]. Studies on cattle at the early stage of MAP infection revealed an upregulation of class I MHC molecules, suggesting a pivotal role of these molecules in the very beginning of the infective process [22]; this is of great interest for both diagnosis and prophylaxis-oriented studies. Figure 1 provides an overview of the major immunological mechanisms triggered byMAP infections.

To date, JD diagnosis relies on both direct (MAP culture, PCR, microarrays etc.) and indirect (ELISA) detection of MAP from feces, milk and necroscopy-derived tissues. However, all the available diagnostic methods suffer from sensitivity (especially in the sub-clinical phase) that strongly reduce their robustness and efficient applicability on large-scale control programs. The failure to detect the subclinical infection makes it difficult to timely apply the control measures necessary to block the spread of the infection within the same, and to other, herds.

A thorough comprehension of the etiopathogenetic mechanisms of MAP infection and host response would be beneficial for diverse research scenarios, providing guidance for the design of MAP-specific diagnostic tools capable of JD diagnosis in the subclinical phase. From this perspective, a previous study from our research group [18] employed an immunoproteomic approach to investigate and select MAP-specific immunoreactive proteins. Here, incubation of MAP proteome with sera from infected bovines highlighted several possible candidate immunoreactive proteins. These candidates represent a good starting point for an immunoinformatic analysis of their sequences in order to find the best immunoreactive sub-sequences and epitopes. This would provide a library of peptides that might be useful for novel prophylactic strategies and/or for their potential application in the immune-based detection of MAP.

The rapid development of the bioinformatics tools and databases provides the possibility to detect the antigenic/epitopic regions of given protein sequences. This innovative strategy for the in-silicoanalysis is time- and cost-effective compared to the “old-fashioned” laboratory-based approach. Recently, Carlos et al. [23] and Rana et al. [24] applied immunoinformatics-based studies to detect class II MHC epitopes possibly useful for the control of JD in a rapid and cost-effective manner.

Over the last decade, these computational approaches lead to the achievement of successful epitopes prediction in several research fields as virology [25,26], bacteriology and cancer research [27].

In this study, previously-selected immunogenic proteins [18] were studied via several immunoinformatics approaches aiming at the detection of the most promising peptide sequences useful for diagnostic purposes. The parameters taken into account were affinity for both the humoral antibody binding and the class I MHC molecule binding. We predicted the most suitable peptide sequences and discuss their potential employment in the design of innovative control measures against JD, with a specific focus on the early diagnosis of JD and/or potential use in novel specific vaccine formulations.

## 2. Results

The peptide sequences of the previously-identified immunoreactive proteins [18] were used to recall the novel protein identifiers in the NCBInr protein database. Because of the continuous evolution of the data repositories and the increasing knowledge on their entries, some protein accession numbers were re-classified into other identifiers. Table 1 summarizes the BLAST-based alignments of the peptides performed to line up the selected proteins to the current identification system. All pBLAST alignments matched at 100% with the reference protein kept. The low E-value of each alignment supports the attribution of the immunoreactive proteins to the novel identifiers.

**Figure 2:**
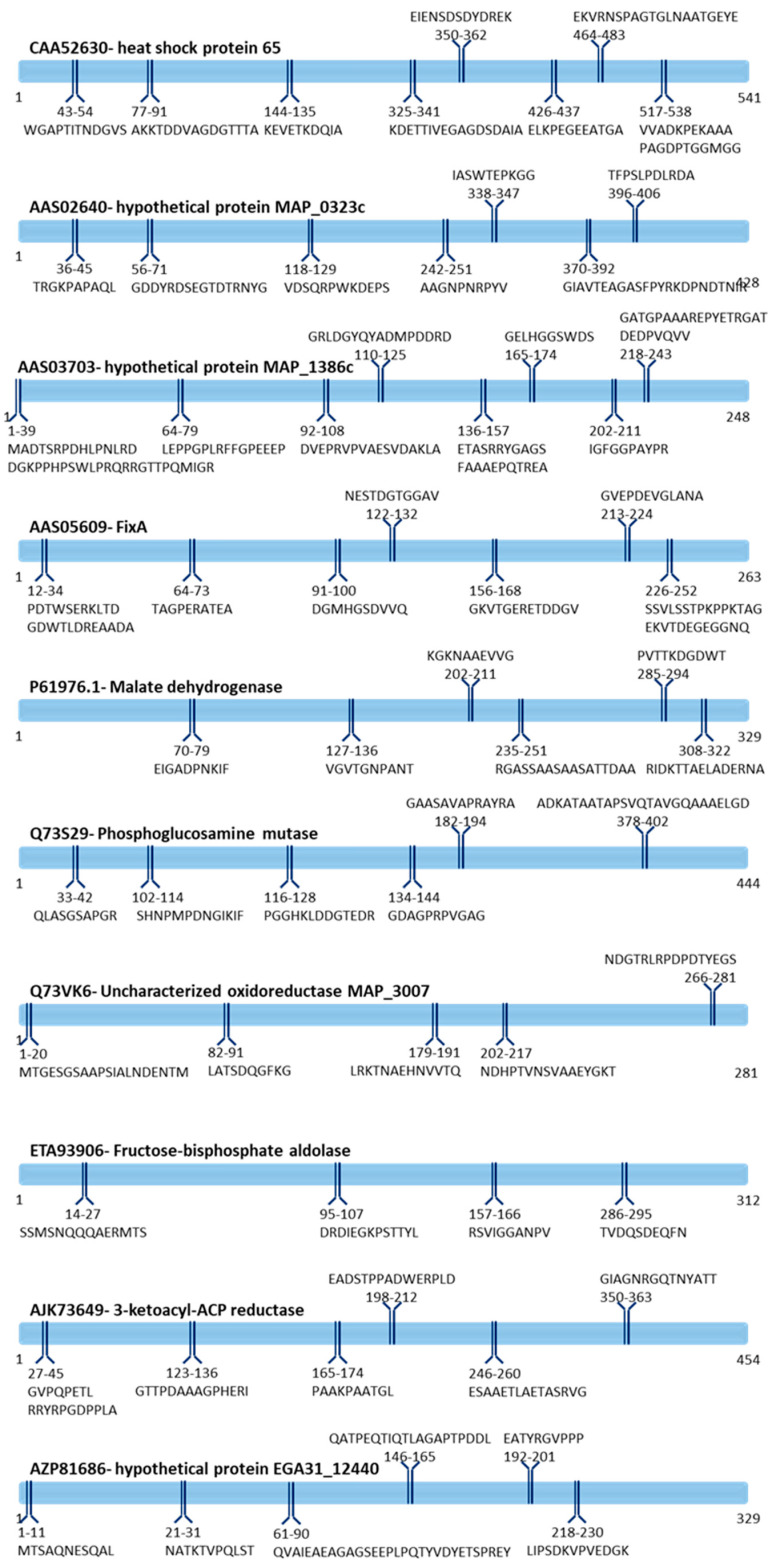
Depiction of the candidate B-cell-binding protein epitopes. The figure represents the selected immunological proteins selected. For each of the protein is displayed the prediction of the B-cell epitopes along with the relative sequence region.

Once the updated protein identifiers are inferred, the major immunogenic domains of the selected proteins were predicted through an immunoinformatic approach. Prediction of the linear B-epitopes provided a list of epitopes capable of eliciting antibody production (Supplementary Table S1). All the selected proteins showed relevant epitopes from an immunogenic point of view. A large number of short epitope sequences is predicted for each immunogenic protein; whereas, an average of six candidate epitopes (min 4-max 8) meeting the threshold of a minimal length of 10 aminoacids is predicted for each of the selected immunogenic proteins. Figure 2 depicts the ten immunogenic proteins of MAP along with the relative distribution of the predicted B-epitopes. Whole protein calculated immunogenic potential based on the type-B epitopes prediction indicates the “hypothetical protein MAP_1386c” (AAS03703) as the most immunogenic one. This evidence is supported by its highest number of predicted epitopes and the highest average epitope length (Figure 2). On the other hand, the fructose-bisphosphate aldolase (ETA93906), reported the lowest number of predicted epitopes along with the lowest epitope sequence length. Regardless of the number of predictions, candidate epitopes are evenly mapped over the full sequence length of the immunogenic proteins, suggesting a good versatility of the predicted sequences (Figure 2).

Prediction of binding affinity for the diverse class I BoLAs histocompatibility antigens predicted a high number of peptide sites. The full list of class I MHC epitope prediction is provided in the Supplementary Table S2. Epitope prediction from the previously selected immunogenic proteins yielded a total of 7044 peptides, each of which scoring a peculiar binding affinity. Peptides scoring a binding affinity among the top 0.5% are considered as strong binders (SB); whereas, peptides with a percentile rank comprised between 0.6% and 2% were labelled as weak binders (WB). Sorting all the entries using the “sole” WB and SB resulted in a total of 818 candidate epitopes when considering all the MHC haplotypes for the ten immunogenic proteins (Supplementary Table S2).

**Figure 3:**
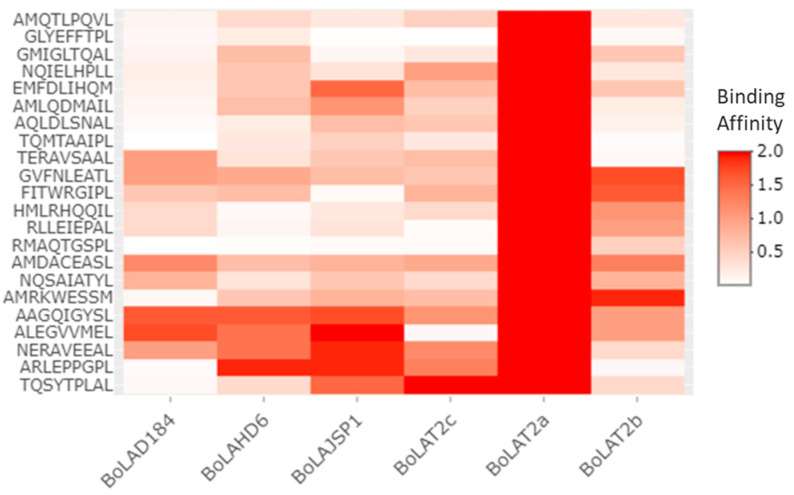
Selected class I MHC binding peptides. The Heat Map displays the selected T-epitopes and summarizes BoLAs haplotypes predicted to bind each sequence as means of a color scale that depends on the binding affinity. Peptides “AMLQDMAIL” belongs to heat shock protein 65 (CAA52630.1); “AQLDLSNAL” and “HMLRHQQIR” belong to hypothetical protein MAP_0323c (AAS02640.1); “ARLEPPGPL” belong to hypothetical protein MAP 1386c (AAS03703.1); “GVFNLEATL” and “NERAVEEAL” belong to the protein FixA (AAS05609.1); “RLLEIEPAL”, “AAGQIGYSL” and “ALEGVVMEL” belong to the Malate dehydrogenase protein (P61976.1); “AMQTLPQVL” AND “RMAQTGSPL” belong to Phosphoglucosamine mutase (Q73S29.1); “NQIELHPLL”, “TERAVSAAL”, “AMDACEASL” and “TQSYTPLAL” belong to Uncharacterized oxidoreductase MAP_3007 (Q73VK6.1); “EMFDLIHQM” and “AMRKWESSM” belong to fructose-bisphosphate aldolase (ETA93906.1); “GLYEFFTPL”, “GMIGLTQAL” and “TQMTAAIPL” belong to 3-ketoacyl-ACP reductase (AJK73649.1); “FITWRGIPL” and “NQSAIATYL” are peptides of the hypothetical protein EGA31_12440 (AZP81686.1).

For a better evaluation of the most suitable MAP epitopes, we focused our attention towards the sequences that are most commonly recognized by the immune system effectors (i.e., BoLA haplotypes and, in turn, CD8^+^ T-cells). Figure 3 lists, for each of the tested immunogenic protein, the shared epitopes among the MHC haplotypes.

The vast majority of the listed epitopes are classified as SB; while eight of them, belonging to the proteins Malate dehydrogenase (P61976), Uncharacterized oxidoreductase MAP_3007 (Q73VK6) and hypothetical protein EGA31_12440 (AZP81686), are to be considered as WB on the basis of their affinity rank. Regardless of the binding affinity, all these sequences are predicted to be commonly bound by a plurality of MHC haplotypes. An average of 2 (min 1-max 4) suitable epitopes are selected for each of the tested protein. Such epitopes are predicted to be recognized by five diverse BoLAs out of the six MHC haplotypes used for the computer-based prediction; except for the immunogenic proteins FixA (AAS05609) whose epitopes can be bound by four BoLAs out of six. The BoLA-HD6, BoLA-JSP.1 and BoLA-T2c are able to recognize all the selected epitopes sequences among the immunogenic proteins. On the other hand, the BoLA-T2a is not showing any binding affinity to the epitope sequences; while BoLA-D18.4 and BoLA-T2b fail to bind the epitopes of AAS05609 protein(Figure 3).

The class I MHC epitopes as of Figure 3 are further aligned against both the mycobacteria and cow databases to assess the specificity of the predicted epitope sequences for MAP. Complete list of alignments is available in the Supplementary Table S3. Sequence alignment highlighted that the peptide AMDACEASL and AMRKWESSM respectively of the “uncharacterized oxidoreductase MAP_3007” (Q73VK6) and “fructose-bisphosphate aldolase” (ETA93906) proteins are the most specific for MAP. Specifically, AMDACEASL scores 100% identity with the MAP and the *Mycobacterium aviumcomplex* (MAC); whereas, hits with other mycobacteria specimens are featured by a lower sequence identity (below 89%) and a far higher E-value when compared with MAP and MAC hits (0.16 vs. 5.3, Supplementary Table S3). Similarly, the peptide AMRKWESSM scores 100% sequence identity with MAP and MAC and only less than 73% of sequence similarity is scored by the alignments with other mycobacteria. The E-value supports the robust alignment against the MAP and MAC (E-value 0.01) in spite of the other alignments (E-value > 86) further supporting the hypothesis on the specificity of this peptide sequence (Supplementary Table S3). Concerning the alignment of the peptides against the cow database, both AMDACEASL and AMRKWESSM did not score relevant matches with any of the cow proteins. Several hits were matching with discontinuous sequences of the cow proteins database with high E-values (Supplementary Table S3, topic better commented in the discussion section).

## 3. Discussion

The host’s immune response to MAP infection is complex and heterogeneous. Debates on the sequelae of immunological events following MAP infection are currently ongoing. Nevertheless, it seems widely accepted that the early stage of the infection elicits an important cell-mediated response. Once MAP is phagocytized, its antigen presentation is accomplished through the loading of the processed antigen onto MHC molecules. The bovine MHC genes complex (i.e., Bovine Leukocyte Antigen, BoLA) is carried in the chromosome 23 and represent a fundamental component of the bovine immune system that allows the recognition and presentation of a virtually infinite number of antigens [28] (Figure 1). Such a high versatility relies on diverse factors, including the polygenetic origin of the MHC genes, the codominance of the parental alleles, the polymorphism of the genetic variants and the peptides/proteins splicing [29]. Class I MHC molecules recognize, bind and present peptide antigens from intracellular pathogens to CD8^+^ T-lymphocytes [28]. In this view, class I MHC molecules and Cytotoxic T-lymphocytes (CTL) are likely to play a pivotal role in the early stage of the MAP infection [30]. Thus, of potential interest for early diagnosis-oriented studies and the design of efficient vaccine formulations. Class I MHC peptide antigens are to be considered among the main triggers of the cell-mediated responseand their specific immunostimulation would lead to a more efficient prophylactic outcome. Nevertheless, a study from Rana etal. highlighted an important involvement also of the humoral response to MAP infection, other than the adaptive immunity mediated by the class II MHC molecules [24].

Huge efforts have been made to optimize diagnostics for the efficient detection of MAP by means of both direct and indirect methods [4,31,32]. The slow-growing rate of MAP along with the reduced sensitivity of the culture-based methods raised the need to develop alternative diagnostic strategies. PCR-based diagnosis targeting the multicopy insertion sequence IS900 held the promise of fast detection of MAP in both environmental and clinical samples. However, the presence of IS900-like sequences in other bacterial specimens resulted in a reduction of the PCR specificity. This, along with the elevated costs of the reagents, equipment and procedures, precludes the PCR applicability in large-scale programs [33].

Among the indirect methods, ELISA-based detection of anti-MAP antibodies enables faster diagnosis time but still suffer from drawbacks related to sensitivity and specificity. Although great improvements have been made in optimizing ELISA kits to reduce cross-reactions with environmental mycobacterium strains [18,34]. Still, this method suffers from a lack of sensitivity. Moreover, the high antigen similarity between MAP and *Mycobacterium bovis* obstacles the discrimination between bovines infected with tuberculosis and inoculated with live or attenuated paratuberculosis vaccines [35]. This promotes the seek of molecular target(s) capable of offering a more robust diagnosis.

The present work describes a companion study that relies on previously-obtained datasets of our research group [18]. Employing an immunoproteomic approach, we experimentally validated the whole MAP proteome for its capability of being complexed by the antibodies naturally occurring in sera of infected bovines. MS-based identification of the immunogenic proteins enabled the detection of 10 protein candidates whose protein sequences have been now further investigated for their immunogenic features. We employed an immunoinformatic approach for further focusing on the peptide sequences, potentially involved in the immunostimulation. A key point of the immunoinformatic approach is the prediction of the protein epitope sequences. Epitopes prediction can be based on several features such as physical-chemical properties and structural folding of the primary protein sequence [36,37,38]. The present investigation is mainly focused on linear epitopes because protein-antibody complexes were selected through two-dimensional electrophoresis (2-DE) and western blotting; thus, on linearized proteins [18,39]. However, the application of other Mass Spectrometry technologies is quickly developing in the field on immunoproteomics [40] there are still significant limitation to map on a large scale conformational epitopes.

As expected from the previous experimental data, all the screened protein sequences showed the capability of being recognized by both B-cells and class I BoLAs. The comprehension of recognition and binding of MAP by the host immune cells is still controversial. Some studies document a relevant humoral response to MAP infections. On the other hand, other pieces of evidence describe the importance of the cell-mediated response to control MAP growth [41,42,43,44,45].

From our perspective and, according to the collected evidence, MAP-targeted antibodies could play a key role in the specific and sensitive detection of this pathogen in the subclinical stage of the infection. B-cell epitopes prediction highlighted the “hypothetical protein MAP_1386c” (AAS03703) protein as the most active in stimulating antibody production. This finding is in agreement with our previous study [18], where this protein showed a high level of immunoreactivity exclusively against the serum of the MAP infected animals. To the best of our knowledge, this protein was not described before as an antigen and, according to our dataset on its functional domains, it is possible to hypothesize that it is part of a surface-associated dehydrogenase with oxidoreductase activity involved in pentose phosphate pathway [18,46]. The fructose-bisphosphate aldolase (ETA93906), instead, is described as less prone to elicit antibody production. This is consistent with its intracytoplasmic localization and with its major role in the central metabolism. Despite its cellular localization, several moonlighting properties have been described as part of its multiple functions [47,48,49]. Interestingly, B-cell epitopes prediction highlighted a homogenous distribution of multiple peptide sequences throughout all the proteins primary sequences (Figure 2). This suggests the potential usefulness of the selected proteins for a variety of implications where two or more epitopes are needed in a single protein molecule (e.g., sandwich ELISA, and other indirect diagnostic tests ensuring higher sensibility) [50,51].

Prediction of the class I BoLAs binding peptides confirmed the immunogenicity of the previously studied proteins. Similarly to HLAs, BoLAs are highly polymorphic proteins; thus, including a plurality of BoLAs while computing the peptide binding affinity would benefit the robustness and reliability of the prediction [52,53]. Among the class I MHC epitopes predicted in the present study, the hypothetical protein EGA31_12440 (AZP81686) differs by only one amino-acidic residue from the MAP membrane protein 2121c (V7KRE0), whose immunogenic properties have been already demonstrated by both our previous investigation and other studies [18,31,54]. It is, indeed, a surface-exposed protein involved in the mechanism of invasion of the epithelial cells [55,56]. Its expression is upregulated when the MAP is exposed to the physicochemical conditions similar to the intestine environment and the specific block of this protein reduces the virulence up to 60% [34]. Interestingly, this protein is among the entries classified as WB suggesting that more immunogenic properties can also be exploited by the other WB protein besides the others predicted as being SBs.

Moreover, we specifically focused on the sole peptide sequences whose binding affinity is shared among multiple BoLAs. In this manner, the most suitable epitope sequences are likely to have a broad recognition in a higher portion of the bovine population [57,58]. Epitopes identified with this approach are of potential interest for diverse purposes and studies, including the investigations aimed at elucidating the order of immunological events following the MAP infection, and shedding light on the controversial aspect of suppression, or not, of the cellular-mediate immune response following MAP infection [3].

To prove selected epitopes as suitable candidates for the unbiased diagnosis of MAP infection, we aligned the peptides sequences against a database comprising the closest taxonomically-related bacteria. Such alignment generated a steep reduction of the number of input sequences and returned two peptides suitable for a specific diagnosis of MAP. These two candidates were not overlapping with other mycobacteria other than *Mycobacterium avium complex* (MAC). The described approach resumes the pipeline of an in-silico method, therefore, empirical tests will be required for the definitive assessment of differential diagnosis capability of the selected sequences. The sequence alignment against the host-specific protein (i.e., the publicly available cow proteome) fails to identify significant sequence identities. The only alignment hits observed (Supplementary Table S3) were not continuously overlapping and showed a low percentage of identity and a high E-value. Acknowledged the prediction of linear epitopes, the matching of our candidates with gapped sequences of cow is likely to be of a negligible relevance since regarding amino acid residues that are not laying in a concatenate order. Thus, we speculate that the candidate epitopes suggested in this study are of potential value for the design of either multi-subunit or recombinant vaccines to confer protection against the first-time infection of the calves by MAP. Nevertheless, confirmatory experimental trials are warmly encouraged especially to assess the specificity between MAP infected animals and bovine with tuberculosis [59,60]. Although less significant, a certain level of identity hasbeen registered with other mycobacterium strains. However, the discrepancy observed in the sequence alignment might be used as the driving force for the differential diagnosis. At this purpose, application of optimized laboratory protocols expecting high stringency condition might be the key to improve the specificity of the diagnostic methods.

Finally, empirical evaluation of the synergistic effect of both B-cell epitopes and the class I MHC epitopes are desirable. This will aim at the evaluation of the successful diagnosis of MAP infection at the subclinical stage and at the potential in elicitation of protective immunity.

To conclude, the present study describes an innovative pipeline based on the in-silico survey of selected immunoreactive proteins capable to uncover the immunogenic features of each protein. This pipeline was applied to the detection of a restricted number of peptides potentially useful for the diagnosis of JD at the early subclinical stage. Obtained results are as well useful for the implementation of innovative vaccination strategies.

The obtained results confirmed the immunogenicity observed experimentally through the immunoproteomic approach applied to the MAP proteome. This evidence demonstrates, once again, reciprocal support between immunoproteomics and immunoinformatics. Nevertheless, empirical confirmations are warmly required to test the provided epitope sequences both in-vitro and ex-vivofor the possible detection of the subclinical phase of the infection and for the efficacy of the eventual vaccinal formulations. Such experimental tests might also help with the comprehension of the controversial role of the host immune cell-response underlying behind JD. Complementation of the linear epitopes array with other conformational ones is also of importance for befitting efficacy and safety of the deliverables Empirical confirmation may serve as further proof of the robustness of the immunoinformatics approaches as key contributors in the study of diverse infectious diseases. This would provide reliable scientific support in a safe, rapid and cost-effective approach.

## 4. Materials and Methods

### 4.1. Data Collection and Protein Sequence Retrieving

The current study focuses on ten proteins whose immunoreactivity has been experimentally investigated by means of an immunoproteomic approach [18]. Brielfy, the MAP proteome was incubated with sera from infected animals to screen for proteins with immunoreactive potential. The most promising entries were then subjected to MS-based identification.

Identifiers of the candidate immunoreactive proteins were queried in the NCBI non-redundant (NCBInr) protein database to retrieve the whole protein sequences and export them as a FASTA file. Update of the protein accession numbers operated by the reference data repository (i.e., NCBI) required the run of a protein sequence alignment for the attribution of the novel protein identifiers (GI numbers). Selected peptide sequences of the immunoreactive proteins were searched against the NCBInr database restricted to *Mycobacterium avium* subsp. *paratuberculosis* (taxID 1770) and the best hit was used to transform the former protein accession numbers into the novel NCBI protein GIs. The list of proteins employed in this study along with their current GI number is provided in Table 1 and Supplementary Table S4.

### 4.2. Epitopes Prediction

Prediction of the protein sequences that are likely to elicit antibody production and/or bind class I MHC proteins was performed through two tools that are commonly employed for the epitope prediction [27], namely IEDB (http://tools.iedb.org/bcell/) and NetMHC (http://www.cbs.dtu.dk/services/NetMHC/), respectively for B- and class I MHC epitopes prediction.

Bepipred algorithm was chosen for the prediction of linear protein epitopes capable of binding B-cells. This employs a combination of a hidden Markov model and a propensity scale method [61]. Each protein residue is scored for its epitope behavior and the sole aminoacid with a score greater than or equal to 0.35 was considered as a potential epitope. Linear peptide epitopes of the least length of 10 aminoacids were selected for this study.

**Figure 4:**
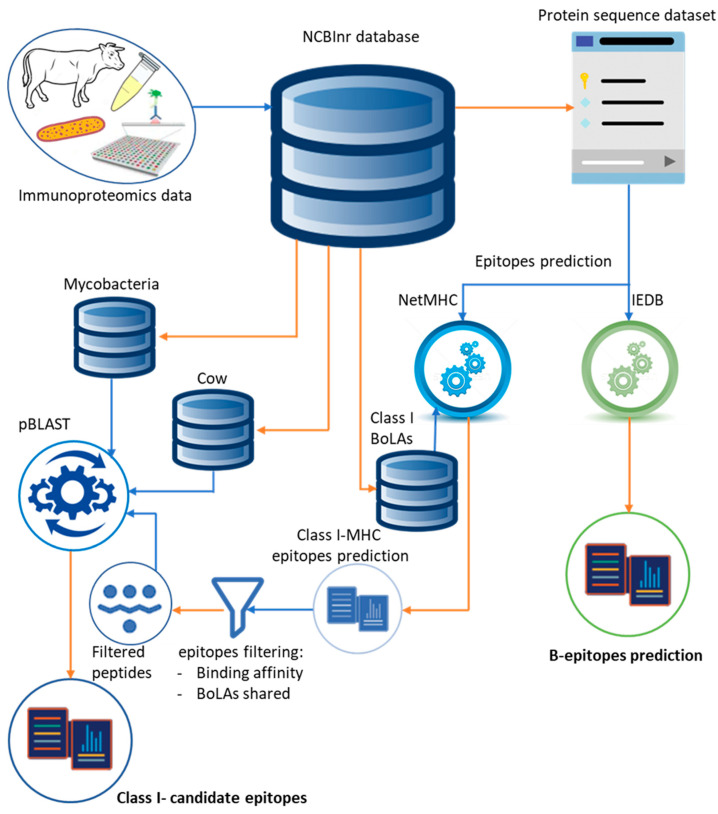
Schematic representation of the immunoinformatic approach. Blue and orange arrows refer to the input and output of the data, respectively. Data arising from our previous immunoproteomic study [18] were used to retrieve the protein sequences of the immunogenic proteins, selected on the basis of their capability of being complexed by the immunoglobulins in the sera of the infected cows. The sequence of the immunogenic proteins is subjected to the epitope prediction through dedicated tools and algorithms. The B-epitope prediction was performed via the IEDB prediction tool, that provides a list of candidate B-epitopes. The class I MHC epitopes waspredicted via NetMHC. This makes use of the BoLA haplotypes from the data repository (NCBI) as references for computing the linear peptides capable of being recognized and presented by the diverse BoLAs. The list of potential T-epitopes is further refined by selecting the most commonly recognized epitopes with a relatively high binding affinity. The refined list of epitopes is further tested for sequence specificity and cross-reactivity by pBLAST alignment versus the mycobacteria and cow protein database which, in turn, arise from the publicly available data repository (NCBI).

Prediction of epitopes binding class I MHC molecules was performed through NetMHC prediction tool, using the artificial neural network (ANN) algorithm [62]. The algorithm was set for the prediction of nine-aminoacids long peptides capable of binding the following BoLA alleles: BoLA-D18.4; BoLA-HD6; BoLA-JSP.1; BoLA-T2a; BoLA-T2b; BoLA-T2c. The binding affinity of the peptides wasscored, and a percentile rank wasprovided by computationally comparing the score of each queried peptide sequence against 400,000 natural peptides of the same length. Peptides scoring a binding affinity up to 0.5% were considered as strong binders (SB); whereas, peptides with a percentile rank comprised between 0.6% and 2% were labelled as weak binders (WB). All other peptides were discarded [3,62,63]. The resulting list of selected peptide epitopes was further quality-checked and filtered. For each of the selected proteins, the epitopes shared among the major number of BoLA haplotypes were kept (i.e., the most commonly recognized in the bovine population), resulting in a consensus list of epitopes to be further used in the study. A summary of the experimental worklow employed in this study is provided in Figure 4.

### 4.3. Epitope Sequences Alignment

The list of epitope sequences was further analyzed through the Basic Local Alignment Search Tool for protein sequences (pBLAST) [64]. This tool implements the PAM30 algorithm to compare protein sequences and calculates the robustness of matches as means of expected values (E-value). This value describes the statistic of matches occurring “by chance”; thus, it decreases exponentially as the score of the match increases.

In the pBLAST, each epitope sequence has been aligned against both mycobacteria (NCBI TaxID 85007) and cow (NCBI TaxID 9913) protein repertoires to evaluate sequence specificity and cross-reactivity (Figure 4), of importance while selecting candidate epitopes to be employed for the effective diagnosis and/or prophylaxis of MAP.

## Figures and Tables

**Table 1:** Peptides alignment and novel protein identifiers attribution.

Former GI	New Accession	Peptide Sequence	Match (%)	Position	E-value
41410034	CAA52630.1	GYISGYFVTDAER	100	196–208	1.58 × 10^−8^
		DETTIVEGAGDSDAIAGR	100	326–343	2.53 × 10^−12^
41408639	P61976.1	LASGSLLGPDRPIELR	100	25–40	1.57 × 10^−10^
		DGDWTIVQGLEIDEFSR	100	290–306	2.71 × 10^−13^
41409105	Q73VK6.1	WNLQLSNAVIFR	100	225–236	5.26 × 10^−8^
		LIDTAAAYGNEAAVGR	100	49–64	2.22 × 10^−10^
41407484	AAS03703.1	MADTSRPDHLPNLR.D	100	1–15	4.69 × 10^−11^
		LGGATGPAAAREPYE	100	216–230	2.08 × 10^−9^
41408219	AZP81686.1	QGVVGLFQPGLVGEQAPGLSVR	100	243–264	2.40 × 10^−16^
		AEAGAGSEEPLPQTYVDYETSPR	100	66–88	2.35 × 10^−18^
41409159	AAS05609.1	EAADAVLDEINER	100	30–42	4.52 × 10^−8^
		DDGMHGSDVVQTGWALAR	100	90–107	8.24 × 10^−14^
41406421	AAS02640.1	DAGLAVTEAGASFPYR	93.75	368–383	2.46 × 10^−9^
41410345	Q73S29.1	YLRHLSK	100	158–164	0.028
41410406	ETA93906.1	IITSPAFTGDR	100	73–83	5.48 × 10^−6^
414109790	AJK73649.1	VVVVGTTPDAAAGPHER	100	119–135	1.68 × 10^−11^
		EHHGGHADILVNNAGITR	100	283–300	2.28 × 10^−13^

## Supplementary Materials

The following are available online at https://www.mdpi.com/2076-0817/9/9/705/s1. **Table S1.** B-cell binding protein epitope prediction. The file summarizes the B-epitope prediction results. Each sheet of the XLS file is relative to one of the ten selected proteins. “Position” column is relative to the position of each aminoachid along the protein sequence; “Residue” indicates the type of aminoacid; “Score” is relative to the epitope propensity attributed by the algorithm and “Assignment” rank each aminoacid residue as epitope or not depending on the prefixed settings. **Table S2.** Class I MHC binding protein epitope prediction. The XLS file summarizes the results in a table reporting: the predicted affinity (nM); the percentile Rank, and the predicted binding Core for all the selected alleles. Two additional columns summarize the predictions across alleles: harmonic mean of the %Rank calculated over all specified alleles (H_Avg_Ranks); the number of alleles covered by a given peptide (N_binders). **Table S3.** Selected peptide epitopes alignment against the mycobacteria and cow databases. The XLS file provides a summary of the pBLAST alignment search. Full description of the table columns and the alignment criteria is available at https://blast.ncbi.nlm.nih.gov/Blast.cgi?PAGE=Proteins. **Table S4.** Full list of the prototypic peptides alignment. The file summarizes the p-BLAST alignment of the two selected epitopes against both the mycobacteria and the cow database. Full description of the table columns and the alignment criteria is available at https://blast.ncbi.nlm.nih.gov/Blast.cgi?PAGE=Proteins.

## References

[1] de Silva K., Plain K., Purdie A., Begg D., Whittington R. (2018). Defining resilience to mycobacterial disease: Characteristics of survivors of ovine paratuberculosis. Vet. Immunol. Immunopathol..

[2] Begg D.J., de Silva K., Di Fiore L., Taylor D.L., Bower K., Zhong L., Kawaji S., Emery D., Whittington R.J. (2010). Experimental infection model for Johne’s disease using a lyophilised, pure culture, seedstock of *Mycobacterium avium* subspecies *paratuberculosis*. Vet. Microbiol..

[3] Purdie A.C., Plain K.M., Begg D.J., de Silva K., Whittington R.J. (2019). Gene expression profiles during subclinical *Mycobacterium avium* subspecies *paratuberculosis* infection in sheep can predict disease outcome. Sci. Rep..

[4] Whittington R., Donat K., Weber M.F., Kelton D., Nielsen S.S., Eisenberg S., Arrigoni N., Juste R., Sáez J.L., Dhand N. (2019). Control of *paratuberculosis*: Who, why and how. A review of 48 countries. BMC Vet. Res..

[5] Windsor P.A. (2015). *Paratuberculosis* in sheep and goats. Vet. Microbiol..

[6] Sweeney R.W., Collins M.T., Koets A.P., Mcguirk S.M., Roussel A.J. (2012). *Paratuberculosis* (Johne’s Disease) in Cattle and Other Susceptible Species. J. Vet. Intern. Med..

[7] Whittington R.J., Reddacliff L.A., Marsh I., McAllister S., Saunders V. (2000). Temporal patterns and quantification of excretion of *Mycobacterium avium* subsp *paratuberculosis* in sheep with Johne’s disease. Aust. Vet. J..

[8] Manning E.J.B., Collins M.T. (2001). *Mycobacterium avium* subsp. *paratuberculosis*: Pathogen, pathogenesis and diagnosis. OIE Rev. Sci. Tech..

[9] Piras C., Soggiu A., Greco V., Alloggio I., Bonizzi L., Roncada P. (2015). Peptidomics in veterinary science: Focus on bovine *paratuberculosis*. Peptidomics.

[10] Wynne J.W., Bull T.J., Seemann T., Bulach D.M., Wagner J., Kirkwood C.D., Michalski W.P. (2011). Exploring the zoonotic potential of *mycobacterium avium* subspecies *paratuberculosis* through comparative genomics. PLoS ONE.

[11] McNees A.L., Markesich D., Zayyani N.R., Graham D.Y. (2015). *Mycobacterium paratuberculosis* as a cause of crohn’s disease. Expert Rev. Gastroenterol. Hepatol..

[12] Pierce E.S. (2018). Could *Mycobacterium avium* subspecies *paratuberculosis* cause Crohn’s disease, ulcerative colitis⋯and colorectal cancer?. Infect. Agents Cancer.

[13] Atreya R., Bülte M., Gerlach G.F., Goethe R., Hornef M.W., Köhler H., Meens J., Möbius P., Roeb E., Weiss S. (2014). Facts, myths and hypotheses on the zoonotic nature of *Mycobacterium avium* subspecies *paratuberculosis*. Int. J. Med. Microbiol..

[14] Feller M., Huwiler K., Stephan R., Altpeter E., Shang A., Furrer H., Pfyffer G.E., Jemmi T., Baumgartner A., Egger M. (2007). *Mycobacterium avium* subspecies *paratuberculosis* and Crohn’s disease: A systematic review and meta-analysis. Lancet Infect. Dis..

[15] Sewell G.W., Marks D.J., Segal A.W. (2009). The immunopathogenesis of Crohn’s disease: A three-stage model. Curr. Opin. Immunol..

[16] Sechi L.A., Dow C.T. (2015). *Mycobacterium avium* ss. paratuberculosis Zoonosis—The Hundred Year War-Beyond Crohn’s Disease. Front. Immunol..

[17] Vaerewijck M.J.M., Huys G., Palomino J.C., Swings J., Portaels F. (2005). Mycobacteria in drinking water distribution systems: Ecology and significance for human health. FEMS Microbiol. Rev..

[18] Piras C., Soggiu A., Bonizzi L., Greco V., Ricchi M., Arrigoni N., Bassols A., Urbani A., Roncada P. (2015). Identification of immunoreactive proteins of *Mycobacterium avium* subsp. paratuberculosis. Proteomics.

[19] Smeed J.A., Watkins C.A., Rhind S.M., Hopkins J. (2007). Differential cytokine gene expression profiles in the three pathological forms of sheep *paratuberculosis*. BMC Vet. Res..

[20] Dennis M.M., Reddacliff L.A., Whittington R.J. (2011). Longitudinal study of clinicopathological features of Johne’s disease in sheep naturally exposed to *Mycobacterium avium* subspecies *paratuberculosis*. Vet. Pathol..

[21] Stabel J.R., Bannantine J.P. (2020). Divergent antigen-specific cellular immune responses during asymptomatic subclinical and clinical states of disease in cows naturally infected with *Mycobacterium avium* subsp. *paratuberculosis*. Infect. Immun..

[22] Purdie A.C., Plain K.M., Begg D.J., de Silva K., Whittington R.J. (2012). Expression of genes associated with the antigen presentation and processing pathway are consistently regulated in early *Mycobacterium avium* subsp. *paratuberculosis* infection. Comp. Immunol. Microbiol. Infect. Dis..

[23] Carlos P., Roupie V., Holbert S., Ascencio F., Huygen K., Gomez-Anduro G., Branger M., Reyes-Becerril M., Angulo C. (2015). In silico epitope analysis of unique and membrane associated proteins from *Mycobacterium avium* subsp. *paratuberculosis* for immunogenicity and vaccine evaluation. J. Theor. Biol..

[24] Rana A., Rub A., Akhter Y. (2015). Proteome-wide B and T cell epitope repertoires in outer membrane proteins of *Mycobacterium avium* subsp. *paratuberculosis* have vaccine and diagnostic relevance: A holistic approach. J. Mol. Recognit..

[25] Tilocca B., Soggiu A., Sanguinetti M., Musella V., Britti D., Bonizzi L., Urbani A., Roncada P. (2020). Comparative computational analysis of SARS-CoV-2 nucleocapsid protein epitopes in taxonomically related coronaviruses. Microbes Infect..

[26] Tilocca B., Soggiu A., Musella V., Britti D., Sanguinetti M., Urbani A., Roncada P. (2020). Molecular basis of COVID-19 relationships in different species: A one health perspective. Microbes Infect..

[27] Raoufi E., Hemmati M., Eftekhari S., Khaksaran K., Mahmodi Z., Farajollahi M.M., Mohsenzadegan M. (2020). Epitope Prediction by Novel Immunoinformatics Approach: A State-of-the-art Review. Int. J. Pept. Res. Ther..

[28] Behl J.D., Verma N.K., Tyagi N., Mishra P., Behl R., Joshi B.K. (2012). The Major Histocompatibility Complex in Bovines: A Review. ISRN Vet. Sci..

[29] Vigneron N., Stroobant V., Chapiro J., Ooms A., Degiovanni G., Morel S., Van Der Bruggen P., Boon T., Van Den Eynde B.J. (2004). An Antigenic Peptide Produced by Peptide Splicing in the Proteasome. Science.

[30] Gyles C.L., Prescott J.F., Songer J.G., Thoen C.O. (2010). Pathogenesis of Bacterial Infections in Animals.

[31] Bannantine J., Stabel J., Lippolis J., Reinhardt T. (2018). Membrane and Cytoplasmic Proteins of *Mycobacterium avium* subspecies *paratuberculosis* that Bind to Novel Monoclonal Antibodies. Microorganisms.

[32] Collins M.T., Lisby G., Moser C., Chicks D., Christensen S., Reichelderfer M., Høiby N., Harms B.A., Thomsen O., Skibsted U. (2000). Results of multiple diagnostic tests for *Mycobacterium avium* subsp. *paratuberculosis* in Patients with inflammatory bowel disease and in controls. J. Clin. Microbiol..

[33] Rathnaiah G., Zinniel D.K., Bannantine J.P., Stabel J.R., Gröhn Y.T., Collins M.T., Barletta R.G. (2017). Pathogenesis, molecular genetics, and genomics of *Mycobacterium avium* subsp. *paratuberculosis*, the etiologic agent of Johne’s disease. Front. Vet. Sci..

[34] Li L., Munir S., Bannantine J.P., Sreevatsan S., Kanjilal S., Kapur V. (2007). Rapid expression of *Mycobacterium avium* subsp. *paratuberculosis* recombinant proteins for antigen discovery. Clin. Vaccine Immunol..

[35] Köhler H., Gyra H., Zimmer K., Dräger K.G., Burkert B., Lemser B., Hausleithner D., Cußler K., Klawonn W., Heß R.G. (2001). Immune reactions in cattle after immunization with a *Mycobacterium paratuberculosis* vaccine and implications for the diagnosis of *M. paratuberculosis* and *M. bovis* infections. J. Vet. Med. Ser. B.

[36] Schönbach C., Ranganathan S., Brusic V. (2008). Immunoinformatics.

[37] Tomar N., De R.K. (2014). Immunoinformatics: A brief review. Methods Mol. Biol..

[38] Backert L., Kohlbacher O. (2015). Immunoinformatics and epitope prediction in the age of genomic medicine. Genome Med..

[39] Lin W.W., Chen I.J., Cheng T.C., Tung Y.C., Chu P.Y., Chuang C.H., Hsieh Y.C., Huang C.C., Wang Y.T., Kao C.H. (2016). A secondary antibody-detecting molecular weight marker with mouse and rabbit IgG Fc linear epitopes for Western Blot analysis. PLoS ONE.

[40] Greco V., Piras C., Pieroni L., Ronci M., Putignani L., Roncada P., Urbani A. (2018). Applications of MALDI-TOF mass spectrometry in clinical proteomics. Expert Rev. Proteom..

[41] Coussens P.M. (2004). Model for immune responses to *Mycobacterium avium* subspecies *paratuberculosis* in cattle. Infect. Immun..

[42] Mortier R.A.R., Barkema H.W., De Buck J. (2015). Susceptibility to and diagnosis of *Mycobacterium avium* subspecies *paratuberculosis* infection in dairy calves: A review. Prev. Vet. Med..

[43] Murphy J.T., Sommer S., Kabara E.A., Verman N., Kuelbs M.A., Saama P., Halgren R., Coussens P.M. (2006). Gene expression profiling of monocyte-derived macrophages following infection with *Mycobacterium avium* subspecies *avium* and *Mycobacterium avium* subspecies *paratuberculosis*. Physiol. Genom..

[44] Koo H.C., Park Y.H., Hamilton M.J., Barrington G.M., Davies C.J., Kim J.B., Dahl J.L., Waters W.R., Davis W.C. (2004). Analysis of the immune response to *Mycobacterium avium* subsp. *paratuberculosis* in experimentally infected calves. Infect. Immun..

[45] Tewari D., Hovingh E., Linscott R., Martel E., Lawrence J., Wolfgang D., Griswold D. (2014). *Mycobacterium avium* subsp. *paratuberculosis* antibody response, fecal shedding, and antibody cross-reactivity to *Mycobacterium bovis* in *M. avium* subsp. *paratuberculosis*-infected cattle herds vaccinated against Johne’s disease. Clin. Vaccine Immunol..

[46] Yum D.Y., Lee Y.P., Pan J.G. (1997). Cloning and expression of a gene cluster encoding three subunits of membrane-bound gluconate dehydrogenase from Erwinia cypripedii ATCC 29267 in *Escherichia coli*. J. Bacteriol..

[47] Zhang C.S., Hawley S.A., Zong Y., Li M., Wang Z., Gray A., Ma T., Cui J., Feng J.W., Zhu M. (2017). Fructose-1,6-bisphosphate and aldolase mediate glucose sensing by AMPK. Nature.

[48] Marsh J.J., Lebherz H.G. (1992). Fructose-bisphosphate aldolases: An evolutionary history. Trends Biochem. Sci..

[49] Tunio S.A., Oldfield N.J., Berry A., Ala’Aldeen D.A.A., Wooldridge K.G., Turner D.P.J. (2010). The moonlighting protein fructose-1, 6-bisphosphate aldolase of Neisseria meningitidis: Surface localization and role in host cell adhesion. Mol. Microbiol..

[50] Aydin S. (2015). A short history, principles, and types of ELISA, and our laboratory experience with peptide/protein analyses using ELISA. Peptides.

[51] Elsohaby I., Mweu M.M., Mahmmod Y.S., McClure J.T., Keefe G.P. (2019). Diagnostic performance of direct and indirect methods for assessing failure of transfer of passive immunity in dairy calves using latent class analysis. Prev. Vet. Med..

[52] Nielsen M., Connelley T., Ternette N. (2018). Improved Prediction of Bovine Leucocyte Antigens (BoLA) Presented Ligands by Use of Mass-Spectrometry-Determined Ligand and in Vitro Binding Data. J. Proteome Res..

[53] Reynisson B., Barra C., Kaabinejadian S., Hildebrand W.H., Peters B., Nielsen M. (2020). Improved prediction of MHC II antigen presentation through integration and motif deconvolution of mass spectrometry MHC eluted ligand data. J. Proteome Res..

[54] Bannantine J.P., Radosevich T.J., Stabel J.R., Berger S., Griffin J.F.T., Paustian M.L. (2007). Production and characterization of monoclonal antibodies against a major membrane protein of *Mycobacterium avium* subsp. *paratuberculosis*. Clin. Vaccine Immunol..

[55] Abdellrazeq G.S., Elnaggar M.M., Bannantine J.P., Park K.T., Souza C.D., Backer B., Hulubei V., Fry L.M., Khaliel S.A., Torky H.A. (2018). A *Mycobacterium avium* subsp. *paratuberculosis* relA deletion mutant and a 35 kDa major membrane protein elicit development of cytotoxic T lymphocytes with ability to kill intracellular bacteria. Vet. Res..

[56] Bannantine J.P., Huntley J.F.J., Miltner E., Stabel J.R., Bermudez L.E. (2003). The *Mycobacterium avium* subsp. *paratuberculosis* 35kDa protein plays a role in invasion of bovine epithelial cells. Microbiology.

[57] Alvarez B., Reynisson B., Barra C., Buus S., Ternette N., Connelley T., Andreatta M., Nielsen M. (2019). NNAlign_MA.; MHC peptidome deconvolution for accurate MHC binding motif characterization and improved t-cell epitope predictions. Mol. Cell. Proteom..

[58] Bazmara S., Shadmani M., Ghasemnejad A., Aghazadeh H., Pooshang Bagheri K. (2019). In silico rational design of a novel tetra-epitope tetanus vaccine with complete population coverage using developed immunoinformatics and surface epitope mapping approaches. Med. Hypotheses.

[59] Hye C.K., Yong H.P., Ahn J., Waters W.R., Hamilton M.J., Barrington G., Mosaad A.A., Palmer M.V., Shin S., Davis W.C. (2004). New latex bead agglutination assay for differential diagnosis of cattle infected with *Mycobacterium bovis* and *Mycobacterium avium* subsp. *paratuberculosis*. Clin. Diagn. Lab. Immunol..

[60] Coetsier C., Vannuffel P., Blondeel N., Denef J.F., Cocito C., Gala J.L. (2000). Duplex PCR for differential identification of *Mycobacterium bovis*, *M. avium*, and *M. avium* subsp. *paratuberculosis* in formalin-fixed paraffin-embedded tissues from cattle. J. Clin. Microbiol..

[61] Larsen J.E.P., Lund O., Nielsen M. (2006). Improved method for predicting linear B-cell epitopes. Immunome Res..

[62] Andreatta M., Nielsen M. (2016). Gapped sequence alignment using artificial neural networks: Application to the MHC class i system. Bioinformatics.

[63] Gurung R.B., Purdie A.C., Begg D.J., Whittington R.J. (2012). In silico identification of epitopes in *Mycobacterium avium* subsp. *paratuberculosis* proteins that were upregulated under stress conditions. Clin. Vaccine Immunol..

[64] Altschul S.F., Wootton J.C., Gertz E.M., Agarwala R., Morgulis A., Schäffer A.A., Yu Y.K. (2005). Protein database searches using compositionally adjusted substitution matrices. FEBS J..

